# Spironolactone Rescues Dot1a-Af9-Mediated Repression of Endothelin-1 and Improves Kidney Injury in Streptozotocin-Induced Diabetic Rats

**DOI:** 10.1371/journal.pone.0047360

**Published:** 2012-10-15

**Authors:** Qiaoling Zhou, Kanghan Liu, Hongyu Wu, Lihe Chen, Veeraragoo Pouranan, Mingxia Yuan, Zhou Xiao, Weisheng Peng, Ao Xiang, Rong Tang, Wenzheng Zhang

**Affiliations:** 1 Department of Nephropathy, Xiangya Hospital, Central South University, Changsha City, Hunan Province, People’s Republic of China; 2 Department of Internal Medicine, University of Texas Medical School at Houston, Houston, Texas, United States of America; 3 Graduate School of Biomedical Sciences, The University of Texas Health Science Center at Houston, Houston, Texas, United States of America; RIKEN Center for Genomic Medicine, Japan

## Abstract

The molecular mechanism linking aldosterone and endothelin-1 in the development of diabetic nephropathy has not been completely elucidated. Here, we provide evidence showing that streptozotocin-induced diabetic rats have significantly increased aldosterone and endothelin-1 in the kidney tissue and markedly decreased expression of Dot1a and Af9. Blocking aldosterone with spironolactone significantly reduced proteinuria, glomerulosclerosis, tubulointerstitial injury and endothelin-1 expression, and significantly increased Dot1a and Af9 expression. Increasing Dot1a and Af9 expression by spironolactone or by stable transfection led to impaired endothelin-1 expression in NRK-52 cells. In contrast, downregulation of Dot1a and Af9 by aldosterone in NRK-52E cells caused upregulation of endothelin-1. Genetic inactivation of *Dot1l*, which encodes Dot1a, in Aqp2-expressing principal cells of mouse kidney impaired association of Dot1a and H3 dimethyl K79 with the specific subregions of endothelin-1 promoter, and upregulates endothelin-1 mRNA and protein expression. Our data suggest that Dot1a and Af9 repress endothelin-1 in vitro and in vivo. Excessive aldosterone induces kidney injury, in part possibly by downregulating Dot1a and Af9, and thus relieving Dot1a-Af9-mediated repression to increase endothelin-1 transcription. Spironolactone ameliorates kidney injury in Streptozotocin-induced diabetic rats, possibly by restoring Dot1a-Af9-mediated repression to reduce endothelin-1 expression. Therefore, Dot1a and Af9 as aldosterone-downregulated targets are negative regulators of endothelin-1 transcription in vitro and in vivo, and may be considered as new potential therapeutic targets of kidney injury in diabetes.

## Introduction

An excessively stimulated renin-angiotensin-aldosterone system has been demonstrated to play a central role in the pathogenesis of diabetic nephropathy (DN). Currently, application of drugs blocking this system is one of the most effective interventions for DN [1]. These inhibitors elicit anti-inflammatory, anti-proliferative and anti-oxidative effects [2]. Spironolactone, a nonselective aldosterone blocker, produces beneficial effects in multiple animal models of renal injury induced by unilateral ureteral obstruction [3], cyclosporin nephrotoxicity [4], hypertension [5] and streptozotocin [6]. Clinical trials suggest that addition of spironolactone to an angiotensin-converting enzyme inhibitor may further improve proteinuria in patients with DN [7]. However, the underlying mechanism is not well defined.

Endothelin-1 (ET-1) is encoded by *Edn-1* gene. In normal physiology, local production and action of ET-1 in the renal medulla promotes diuresis and natriuresis. In pathology, ET-1 promotes vasoconstriction, mesangial cell proliferation, extracellular matrix production and inflammation, and mediates these effects in an autocrine/paracrine manner [8]. Various stimuli including mechanical strain [9], shear stress [10]; oleic acid [11], angiotensin and vasopressin [12], and high glucose [13] stimulate ET-1 expression.

Histone methyltransferases (HMTs) consists of three families: the suppressor of variegation, enhancer of zeste, and trithorax domain family of lysine HMTs, the protein arginine methyltransferase family of arginine HMTs, and the Dot1 (disruptor of telomeric silencing) family. *Dot1* was originally cloned in a yeast genetic screen for genes affecting telomeric silencing [14]. *Dot1* and its human and mouse homologs (*DOT1L* and *Dot1l*) subsequently were found to encode a methyltransferase. Unlike the other two types of HMTs that methylate the NH2 terminal tails of histones, Dot1 specifically targets histone H3 K79, which resides in the globular domain [15], [16], [17]. Histone H3 K79 hypermethylation was observed at the recombination-active rather than at the inactive loci in mammalian cells, and has been considered to be a conserved hallmark of active chromatin regions [18]. *Dot1l* encodes five alternative splicing variants (Dot1a-e) [17] and is essential in embryonic development [19], hematopoiesis [20], [21], cardiac function [22], and the development of leukemia [21], [23], [24].

Characterization of human leukemia samples containing a t(9,11)(p22;q23) translocation led to identification of AF9 [25]. Mouse Af9 is abundantly and widely expressed in kidney and multiple other tissues [26], [27]. There are more than 30 genes known to generate in-frame fusion proteins with the mixed lineage leukemia (MLL). These fusion proteins are thought to cause leukemogenesis [28]. MLL-AF9 is one of the most common forms [29]. AF9 interacts with several proteins including AF4, another MLL fusion partner [30], specific isoforms of BCL-6 corepressor (BCoR) [29], and MPc3, a member of the Polycomb group multiprotein complexes involved in gene silencing by modifying chromatin structure [27]. Apart from these observations, little is known about the AF9 function in normal cells.

We have previously reported a new aldosterone-signaling network regulating the transcription of the epithelial Na^+^ channel subunit α gene (αENaC), using mouse inner medullary collecting duct IMCD3 cells and kidneys of Sgk1 WT and mutant mice as model systems. In this network, Dot1a [31] and Af9 [32] form a protein complex that represses *αENaC* in an aldosterone-sensitive manner. Under basal conditions, Dot1a-Af9 binds to the specific subregions of *αENaC* promoter, promotes H3 K79 methylation, and inhibits transcription [31], [32]. Aldosterone relieves the repression by decreasing mRNA expression of Dot1a and Af9, and by impairing Dot1a-Af9 interaction through Sgk1-mediated Af9 phosphorylation at Ser435 [33]. Hence, transcriptional activation of α*ENaC* by aldosterone can be partially attributed to induction of Sgk1 and downregulation of Dot1a and Af9 mRNA expression. Similar mechanisms may be applicable to regulation of *Edn-1*, since Af9 overexpression significantly decreases ET-1 mRNA expression in 293T and IMCD3 cells [32], [34]. However, whether *Edn-1* is repressed by Dot1a-Af9 in vivo and if such regulation is contributory to spironolactone-mediated improvement of DN has never been addressed. Accordingly, we use streptozotocin (STZ)-induced diabetic rats, mice with Dot1a genetically deleted in Aqp2-expressing connecting tube/collecting duct principal cells, and cultured NRK-52E cells as model systems to address these questions. Our data suggest that Dot1a-Af9 represses *Edn-1* in vitro and in vivo, and spironolactone improves kindney injury in STZ-induced diabetic rats, possibly in part by restoring Dot1a-Af9-mediated repression to inhibit *Edn-1* transcription. Therefore, the current study suggests a novel network linking the spironolactone action and its beneficial effect on kidney injury to transcriptional control of *Edn-1* through modulation of Dot1a and Af9.

## Materials and Methods

### Reagents

Rabbit polyclonal anti-ET-1 (American Research Products, Belmont, MA, USA), anti-Af9 (Bethyl Laboratory, Montgomery, TX, USA), anti-di-methyl H3 K79 (H3m2K79, abcam), and anti-Dot1l (abcam and Bethyl) antibodies, mouse monoclonal anti-CD63 for ED-1 (abcam), spironolactone (Pharmacia Corp., Zhejiang, China), aldosterone, and streptozotocin (Sigma-Aldrich Co., St. Louis, MO, USA), MTT Kit (Chemicon, CA, USA), Chromatin Immunoprecipitation Kit (Millipore) were used according to the manufacturer’s instructions. NRK-52E cells were kindly provided by Xueqing Yu (Sun Yat-sen University, Guangdong, China). pcDNA3.1 and its derivatives expressing untagged Af9 and Dot1a were previously described [31], [32].

### Cells Culture and Stable Transfection

NRK-52E cells were cultured at 37°C in 5% CO2-95% air in Dulbecco’s modified Eagle’s medium supplemented with 10% fetal calf serum, 2% each of penicillin and streptomycin, 1% HEPES, 2 g sodium bicarbonate, and 2 mM L-glutamine. In some experiments, cells were serum-starved for 24 h, and then treated with aldosterone at various final concentrations for 24–72 h as indicated in the Fig. legends. In the experiments involving use of spironolactone, cells were pretreated with 100 nM spironolactone for 1 h before addition of 50 nM aldosterone. To establish stable cells, NRK-52 cells were transfected with pcDNA3.1 as vector control or its derivatives expressing untagged Af9 or Dot1a, using FuGENE 6 (Roche, Indianapolis, USA) according to the manufacturer’s instruction. Forty-eight hours after the transfection, cells were selected with G418 (800 µg/L) for 3 weeks, with medium changed every other day. Surviving colonies from each transfection were pooled and analyzed for expression of Dot1a, Af9, and ET-1 by immunoblot.

IMCD3 cells were seeded and cultured with DMEM/F12 plus 10% FBS for 24 h, then switched to DMEM/F12 plus 10% charcoal-stripped FBS for 53 h. Cells were split and starved for another 16 h in the same medium, treated with 1 µM aldosterone or 0.1% ethanol as vehicle control for 24 h, and then harvested.

### Cell Viability Assay

Briefly, cells were seeded in 96-well tissue culture plates overnight and made quiescent in serum-deprived conditions for 24 h. Cells were then cultured with or without different concentrations of aldosterone (0.01–100 nM) for 24–72 h. MTT solution (5 mg/ml) was added to the wells (10 µl/well). After incubation at 37°C for 4 h, acid-isopropanol (0.04 N HCl in isopropanol, 100 µl/well) was added and mixed thoroughly to each well. Absorbance of each sample was determined at 570 nm.

### Semi-quantitative RT-PCR, Real-time RT-qPCR, and Chromatin Immunoprecipitation (ChIP)

The semi-quantitative reverse-transcript-PCR (RT-PCR) was performed similar to as described previously [23]. Briefly, total RNAs were pretreated with DNase I to eliminate potential genomic contamination, and verified by RT-PCR in which the reverse transcriptase was not added. cDNA was then generated with iScript cDNA Synthesis kit (Bio-Rad) and amplified with primers specific for rat Dot1a, Af9, or ET-1 in separate wells. To control for variability, a pair of primers specific for GAPDH or β-actin was included in each PCR reaction to serve as internal control. The relative intensity of each target band was quantified using ImageJ64 and normalized to that of the internal control. Real time RT-qPCR and ChIP were conducted as described previously [31], [35], [36], with minor modifications. In brief, after optimization of the PCR conditions for each pair of primers, real-time RT-qPCR with the cDNA prepared as above or qPCR with DNA isolated from immunoprecipitated chromatin were conducted on Stratagene MX3000P (Agilent Technologies) with 0.5XSYBR Green Supermix (Bio-Rad). Each sample was amplified in triplicate. The copy number of each sample was calculated using the 2^-ΔΔCt^ method [37], according to threshold cycle value, measured from cycle-dependent product amplification curves. The copy numbers of Dot1a and ET-1 transcripts were normalized to that of β-actin internal control from the same sample. For ChIP, the relative Dot1a or dimethylated histone H3 K79 at each subregion was determined by measuring the apparent immunoprecipitation efficiency (the ratio of the copy number of the ChIP sample to that of the corresponding input). The sequences of all primers are listed in Table 1.

**Table 1:** Sequences of primers for RT-PCR, real-time RT-qPCR, and ChIP.

Gene	Sequence (5′→3′)	Length (bp)
Rat ET-1	F: CACCTGGACATCATCTGG	114
	R: GTCTGTGGTCTTTGTGGG	
Rat Dot1a	F: CAAAACTCAGGGAGGAGCAG	264
	R: TGGCAGCACTCATTTTCTTG	
Rat Af9	F: CCGCTTTGATTATGACTT	420
	R: TCTTTGGGTTTCTTGGAG	
Rat β-actin	F: GACCGAGCGTGGCTACAGC	340
	R: TCTCAGCGATGCCTGGCTAC	
Rat GAPDH	F: CTACCCACGGCAAGTTCAAT	111
	R: GGATGCAGGGATGAT	
Mouse Dot1a	F: CAACAGCAGGAACTTGAGTGACATTGGC	515
	R:ACGCATCCTGGGGTGAGGCTGAGGG	
Mouse ET-1	F: TTGTTCAGACGGGCAGAGGACCAGC	524
	R: CGGAGAGGAGAGAATACAGGGAGG	
Mouse β-actin	F: GTGGGCCGCCCTAGGCACCA	540
	R: CTCTTTGATGTCACGCACGATTTC	
Subregion A	F: GGCTGAGGTAAGCAAAGCAGAG	489
	R: GCAAAACACACAGAAAACACG	
Subregion B	F: CGTGTTTTCTGTGTGTTTTGC	497
	R: TATTTCTTGTGCCCAGCCTCTC	
Subregion C	F: GAGAGGCTGGGCACAAGAAATA	531
	R: GCCCCAGAGTTCAAGAATCAAG	
Subregion D	F: CTTGATTCTTGAACTCTGGGGC	578
	R: GTCAGAAGAGTGGGGAAAAAGG	

F: Forward. R: Reverse. Subregions A–D: 5′ flanking region of mouse Edn-1.

### Immunofluorescence

The mouse kidney tissues were fixed in 4% paraformaldehyde overnight at 4°C and embedded in paraffin. After boiling in antigen-retrieval buffer, paraffin sections were blocked with 5% BSA/0.5% Triton X-100 in PBS. Anti-di-methyl H3 K79 and anti-CD63 were diluted in 5% BSA/0.5% Triton X-100 in PBS and incubated overnight at 4°C. Following 4×5-minute washes in PBS, the sections were incubated with Alexa Fluor 488–conjugated goat anti-rabbit IgG and Alexa 594-conjugated goat anti-mouse IgG (Invitrogen), respectively. DAPI was used to visualize nuclei. The sections were mounted in VECTASHIELD HardSet Mounting Medium (H-1400, VECTOR LABROTORIES), and examined under an epifluorescence microscope (Olympus IX71) as previously described [26], [35].

NRK-52E cells were fixed with 4% freshly prepared paraformaldehyde for 2 h, washed with PBS 3×5 min, and incubated in 3% H_2_O_2_ for 10 min. After 3×5 min washing with PBS, cells were blocked with normal goat serum for 30 min, and then labeled with rabbit antibodies specific for Af9 (1∶200) or ET-1 (1∶200) for 2 h at 37°C or overnight at 4°C. Following 4×5 min washes in PBS, cells were incubated with TRITC or FITC-conjugated goat anti-rabbit IgG for 1 h at 37°C. The cells were mounted in 50% glycerol in 20 mM phosphate buffer (pH 8.5) medium.

### Animals

Male Wistar-albino rats at age 8–10 weeks and initial body weight of 180–220 g were used. Rats were housed at a constant ambient temperature (∼22°C) under a 12-h light cycle. All rats were fed rat chow ad libitum, with free access to water. Diabetes (n = 16 rats) was induced by a single, intraperitoneal dose of freshly prepared STZ (60 mg/kg body weight) in 0.1 M citrate-phosphate buffer (pH 4–5) whereas normal control (n = 8 rats, CT) received an equal volume of buffer per kg body weight. Four weeks after the injection, STZ-injected animals received either no treatment (n = 8 rats, STZ) or 40 mg/kg/day of spironolactone by intragastric administration for 4 weeks (n = 8 rats, SP). At the end of the experiment, systolic blood pressures were measured using tail-cuff plethysmography. Urinary creatinine concentrations were determined by a modified Jaffe method. Urinary protein levels were measured by nephelometry and normalized to urinary creatinine concentration. Plasma glucose levels were assessed by a glucose oxidase method. Plasma and tissue aldosterone concentrations were obtained by a radioimmunoassay kit (Coat-A-Counts kit, DPC, Los Angeles, CA, USA) [36], [38]. Animals were anesthetized by intraperitoneal injection of sodium pentobarbital (50 mg/kg body weight) and then sacrificed by cervical dislocation. Kidneys were removed, weighed, and then either frozen at −80°C until use or fixed according to our published protocol [36]. Generation and characterization of *Dot1l^f/f^* [24] and *Dot1l*-deficient mice *Dot1l^AC^* using *Aqp2:Cre* line [39] have been briefly described (Wu et al, 2011 ASN abstract # 21278 and Wu et al, 2011 ASN abstract 20636) and will be detailed elsewhere.

Diabetic rat experiments were conducted in accordance with Central South University Guide for Laboratory Animal Use, and were approved by Central South University. Animal studies involving use of *Dot1l^f/f^* and *Dot1l^AC^* mice were performed in accordance with NIH Guides for the Care and Use of Laboratory Animals approved by the University of Texas Health Science Center at Houston Animal Welfare Committee (Permit Number: 10-024).

### Immunoblotting, Immunohistochemistry and Metabolic Balance Studies

These assays were conducted according to our published protocols [31], [32], [36]. A semi-quantitative score for a tubulointerstitial injury index was used to evaluate the degree of tubulointerstitial injury on Masson-stained sections. The severity of tubulointerstitial injury was graded from 0 to 4 as follows: grade 0, no obvious change; grade 1, lesions involving less than 25% of the area; grade 2, lesions affecting 25–50%; grade 3, lesions involving more than 50%, and grade 4, involving almost the entire area. Ten microscope fields were analyzed in each kidney section from each rat. Each rat was represented by the average score of these fields [40]. The number of ED-1-positive cells was counted in at least 20 glomeruli and 20 fields of the tubulointerstitium/section under ×200 magnification.

### Statistical Analysis

Quantitative data are presented as mean±SEM and analyzed by Student t test or ANOVA for multiple group comparisons, using a microcomputer-assisted program SPSS for Windows 11.5 (SPSS Inc., Chicago, IL, USA). The statistical significance is set at P<0.05.

## Results

### Spironolactone Improves Proteinuria, Glomerulosclerosis, and Tubulointerstitial Injury in STZ-induced Diabetic Rats

**Figure 1:**
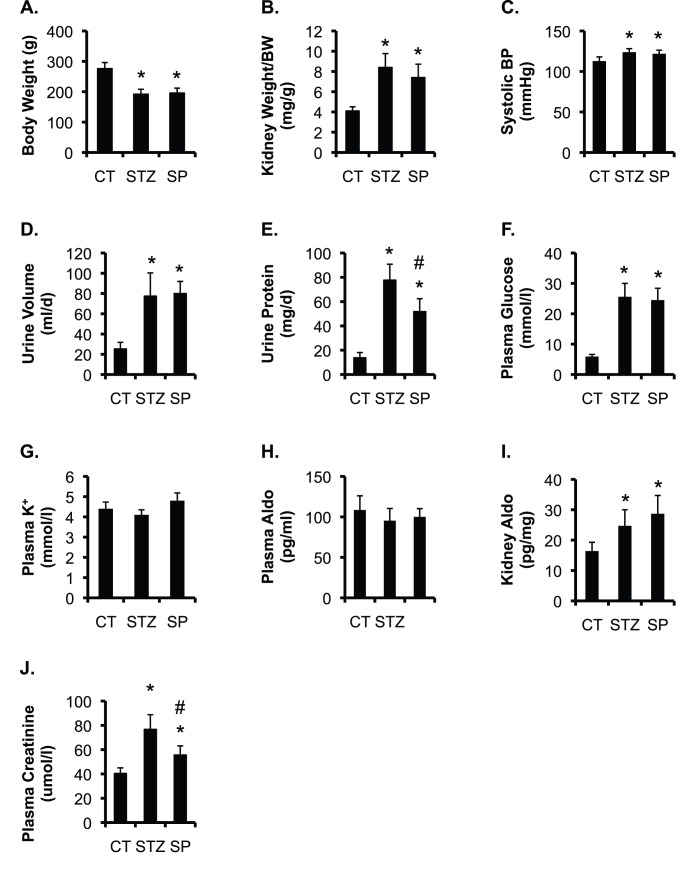
Spironolactone significantly decreases urinary protein excretion and plasma creatinine concentrations in STZ-induced diabetic rats. Control rats (CT), rats received one injection of STZ (60 mg/kg BW) and were allowed to develop kidney damage for 8 weeks (STZ). STZ rats received one injection of STZ (60 mg/kg BW) and were allowed to develop kidney damage for 4 weeks, and then treated with spironolactone (40 mg/kg/day, SP) for 4 weeks, and were analyzed at the end of experiment for the parameters as indicated. n  = 8 rats/group. In all cases, *P<0.05 vs. CT. #P<0.05 vs. STZ. Aldo: aldosterone.

To determine the beneficial effect of spironolactone on kidney injury in STZ-induced diabetic animal model, rats were divided into three groups: control (CT), STZ alone (STZ), and STZ + spironolactone (SP) (n = 8 rats/group, see Methods). The urinary and plasma parameters from these animals were measured at the end of the experiments and are shown in Fig. 1. Body weight was significantly decreased in STZ and SP vs. CT. Kidney/body weight ratio, systolic BP, urine volume, urinary protein excretion, [aldosterone] in kidney tissue, and plasma [glucose] were significantly higher in STZ and SP than in CT. Plasma [K^+^] and plasma [aldosterone] were statistically indistinguishable among the groups. However, spironolactone markedly lowered urinary protein excretion, and had no obvious effect on all other urinary and plasma parameters examined in SP vs. STZ rats (Fig. 1). These observations suggest that the beneficial effect of spironolactone is most likely independent of hemodynamic mechanisms and that spironolactone apparently has little impact on aldosterone metabolism. The major renal response to spironolactone is thus assumed to be through mineralocorticoid receptors, as suggested by others (reviewed in [41]).

**Figure 2:**
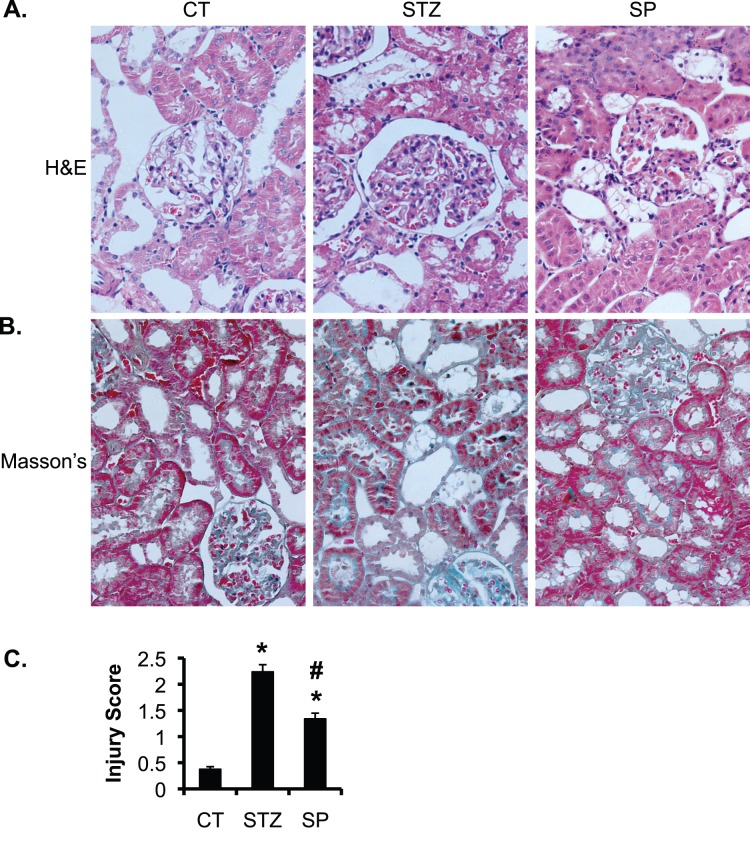
Spironolactone significantly improves glomerulosclerosis and tubulointerstitial injury in STZ-induced diabetic rats. (**A–B**) As in Fig. 1, representative renal histological images showing hematoxylin and eosin stain (**A**) and Masson’s Trichrome stain (**B**) of the rats in the three groups as indicated. (**C**) Whole kidney average score of tubulointerstitial injury based on Masson’s Trichrome stain. n = 8 rats/group. *P<0.05 vs. CT. #P<0.05 vs. STZ.

Fig. 2 shows representative images of hematoxylin/eosin staining and Masson’s trichrome staining. Compared to the normal renal histology in CT, STZ rats exhibited severe glomerulosclerosis and tubulointerstitial injury. The injury is characterized by vacuolated or detached renal tubular epithelial cells, and markedly dilated capillary loops. Glomerular diameters and accumulation of the extracellular matrix in mesangial areas were all significantly increased. These observations are in agreement with previous reports [6], [42] showing that STZ induced kidney damage in rats three weeks after its administration (See Discussion). Spironolactone significantly improved the phenotypes in SP vs. STZ. The overt improvement includes relieved mesangial matrix’s hyperplasia and alleviated tubular epithelial cell degeneration. The tubulointerstitial injury score based on Masson’s Trichrome staining was elevated from 0.375 in CT to 2.238 in STZ, and reduced to 1.338 in SP rats (Fig. 2).

### Spironolactone Significantly Reduced the Number of Glomerular and Tubulointerstitial Macrophages in STZ-induced Diabetic Rats

**Figure 3:**
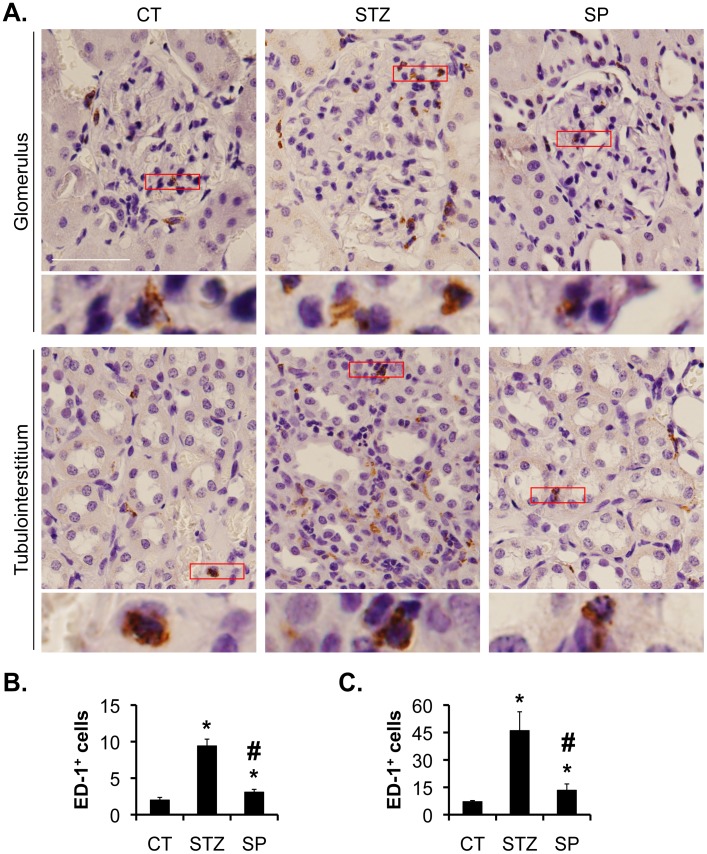
Spironolactone reduces the numbers of glomerular and tubulointerstitial macrophages in STZ-induced diabetic rats. (A) Representative IHC images showing the staining of CD-1, a marker of macrophage, in the kidneys as indicated (A). Boxed areas are amplified and shown at the bottom of each panel. Scale bar: 100 µm and 25 µm for the amplified boxed areas. (B–C) Graphs showing significantly more glomerular (B) and intersterial (C) ED-1-positive cells in STZ vs. CT rats. SP significantly reduced the number of glomerular and tubulointerstitial macrophages in STZ rats.

With ED-1 as a marker of macrophages, we assessed the presence of macrophages by IHC with an antibody specific for ED-1 (Fig. 3A). This antibody has been used by others [43], [44], [45]. The numbers of glomerular and tubulointerstitial macrophages were significantly higher in STZ vs. control (9.4±0.9 vs. 2±0.4 and 46.1±10.3 vs. 7.1±0.6), and Spironolactone treatment significantly decreased the number of ED-1^+^ cells in SP rats (3.1±0.4, and 13.4±3.5) (Fig. 3, B–C). The numbers of glomerular and tubulointerstitial macrophages in the CT and STZ groups are comparable with those reported by others [46].

### Spironolactone Partially Normalized the Expression of Dot1a, Af9 and ET-1 in STZ-Induced Diabetic Rats

**Figure 4:**
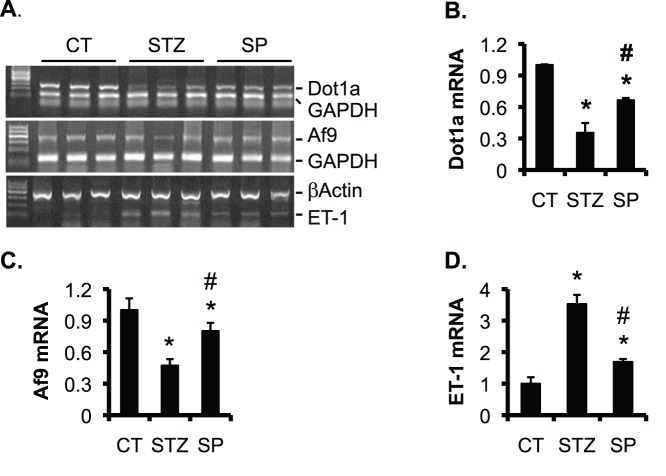
Spironolactone partially normalizes mRNA expression of Dot1a, Af9, and ET-1 in STZ-induced diabetic rats. (A) As in Fig. 1, representative agarose gel analyses of RT-PCR for expression of genes as indicated in the cortex of rats. (**B–D**) For each mRNA as indicated, the intensity of each corresponding band was quantified using ImageJ64, normalized to that of GAPDH or β-actin as shown in **A**, averaged within each group, and plotted, with the relative intensity in control (CT) set to 1. n = 8 rats/group. *P<0.05 vs. CT. #P<0.05 vs. STZ.

The improved kidney function by spironolactone suggests a pathological role of aldosterone in the development of kidney injury in STZ-induced diabetic rats. To identify the underlying molecular mechanism, we focused on Dot1a, Af9, and ET-1, all of which are regulated by aldosterone in IMCD3 cells, as shown by others and us [26], [31], [32], [33], [47]. We isolated the total RNA of the renal cortex from the three groups (n = 8 rats/group) and performed similar semi-quantitative RT-PCR analysis as described [23]. Representative agarose gel analyses of the final RT-PCR products were shown in Fig. 4A. Dot1a and Af9 mRNA levels were significantly decreased to 35% and 47% of CT in STZ rats, and restored to 67% and 80% of CT in SP rats, respectively (Fig. 4 B–C). In contrast, ET-1 mRNA was elevated to 352% of CT in STZ, and diminished to 168% of CT in SP rats (Fig. 4D).

**Figure 5:**
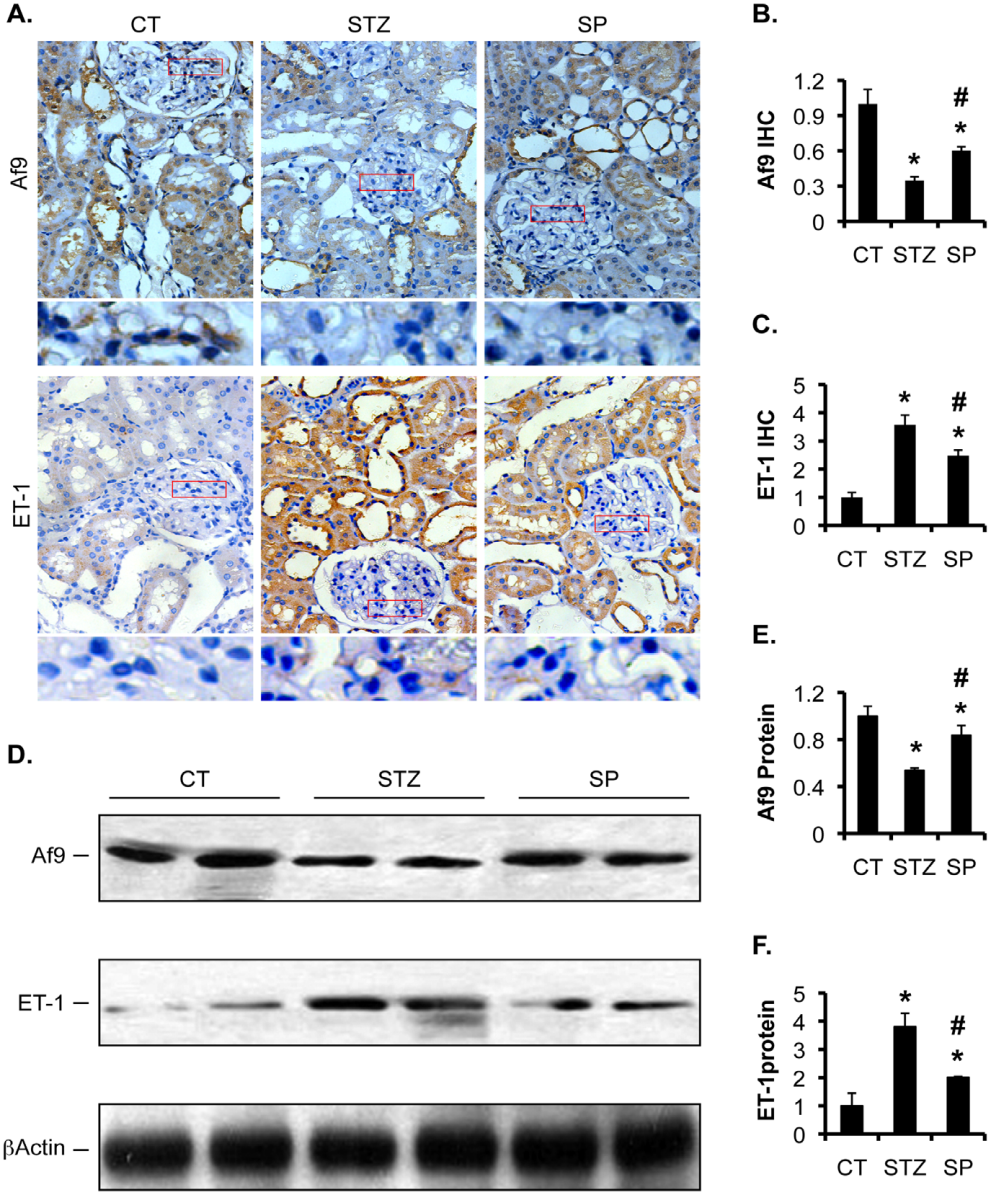
Spironolactone partially normalized protein expression of Af9 and ET-1 in STZ-induced diabetic rats. (**A**) As in Fig. 1, representative IHC images showing expression of Af9 and ET-1 in kidneys of rats as indicated. Boxed areas in the glomeruli were amplified and shown at the bottom of each panel (**B–C**) IHC intensity in **A** was quantified using ImageJ64, averaged within each group, and plotted as indicated, with the relative intensities in controls (CT) set to 1. (**D**) Representative IBs showing expression of Af9, ET-1, and β-actin as loading control in the cortex of rat kidneys as indicated. (**E–F**) The intensity of each band in **D** was quantified using ImageJ64, normalized to that of β-actin, and plotted as indicated, with the relative intensities in controls (CT) set to 1. In all cases, n = 8 rats/group. *P<0.05 vs. CT. #P<0.05 vs. DN.

Af9 and ET-1 protein expression were also changed accordingly. We have previously reported that Af9 is widely detected by immunofluorescence staining in mouse kidney. Af9 staining was found in Aqp2^-^ and Aqp2^+^ tubular cells [26]. As shown in Fig. 5A, immunohistochemistry (IHC) with the same Af9 antibody revealed that control rats expressed robust Af9 in the proximal and distal tubular epithelia, Bowman's capsule-parietal layer, and some intraglomerular cells. In STZ group, Af9 expression was substantially weakened in the proximal and distal tubular cells. It became nearly undetectable in the parietal layer and in the intraglomerular cells. Compared to STZ rats, SP rats had partially rescued Af9 expression in the tubular epithelia and in the parietal layer, but maintained very low or no Af9 expression inside the glomeruli. However, ET-1 IHC staining was very faint in the proximal and distal tubules and negligible inside glomeruli in control rats. STZ administration induced significantly higher ET-1 expression in the tubular epithelia. STZ treatment also led to clearly noticeable ET-1 staining in the intraglomerular cells, although the effect was much less pronounced than in the tubular epithelia. Spironolactone attenuated ET-1 staining to an intermediate level throughout the kidney (Fig. 5A). Quantification of the IHC staining revealed that STZ rats had decreased Af9 to 34% of CT and increased ET-1 to 357% of CT. Spironolactone significantly reversed these numbers to 60% and 247%, respectively (Fig. 5B–C). Consistently, immunoblotting analyses (IB) confirmed these findings. Af9 was markedly decreased to 53% of CT and ET-1 was upregulated to 381% of CT in STZ animals. Spironolactone partially rescued them to 84% and 200%, respectively, in SP rats.

We were unable to evaluate Dot1a protein levels because all of the Dot1a antibodies tested did not specifically recognize rat Dot1a in IHC and IB. Similar technical challenges apparently prevented others from showing disruption of Dot1a expression in *Dot1l*-deficient embryos [19] and in the heart of the cardiac-specific *Dot1l* knockout mice [22]. In fact, to our knowledge, there have been no reports showing expression and distribution of Dot1a and its counterparts by IHC or immunofluorescence in any species including mouse and human.

**Figure 6:**
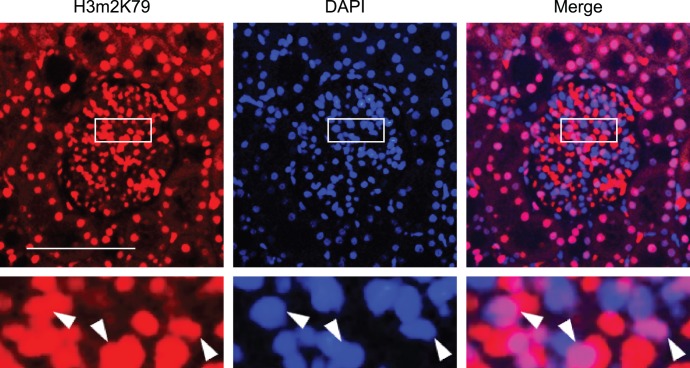
Dot1a as indicated by H3m2K79 is expressed in glomerular cells. Representative immunofluorescence images showing di-methyl histone H3 K79 (H3m2K79) staining in a normal rat glomerulus. Boxed areas were amplified and shown at the bottom. H3m2K79, an indicator of Dot1a methyltransferase activity, was stained in red. Nuclei were visualized by DAPI staining. The merged images demonstrate the presence of H3m2K79 in the nuclei of some (arrowhead), but not all of cells in the glomerulus. Scale bar: 100 µm and 25 µm for the amplified boxed areas.

*Dot1ll* has been shown to be solely responsible for histone H3 K79 di-methylation (H3m2K79) in mice by others [19], [22] and us [24]. We used H3m2K79 as an indicator of Dot1a methyltransferase activity to determine Dot1a expression in glomeruli of normal rats. Immunofluorescence experiments with an antibody against H3m2K79 revealed that some, but not all of glomerular cells displayed strong nuclear staining (Fig. 6). Others and we have confirmed the specificity of the anti-H3m2K79 [19], [22], [24]. Accordingly, Dot1a is most likely expressed in the glomeruli.

### Aldosterone Decreases Dot1a and Af9 and Increases ET-1 Expression in NRK-52E Cells

**Figure 7:**
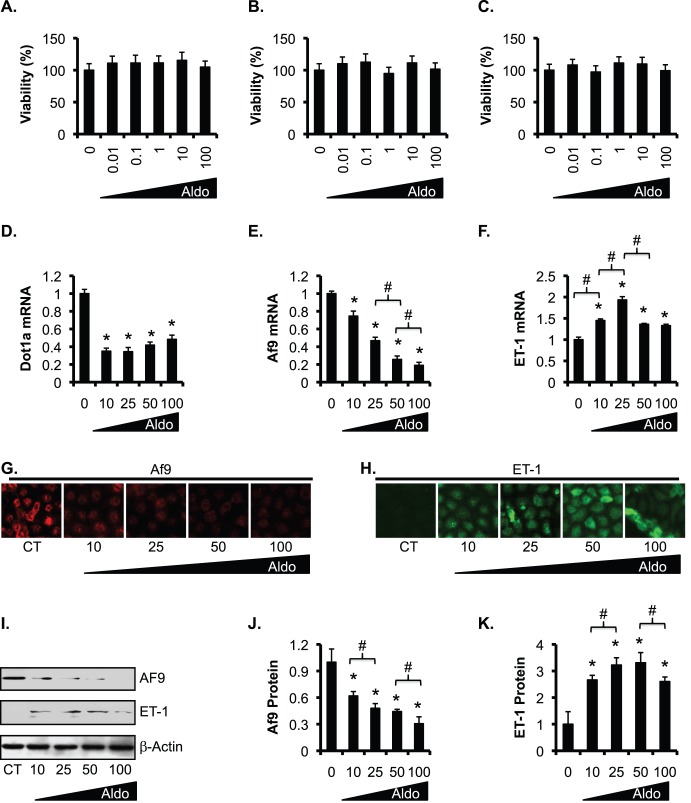
Complex dosage effects of aldosterone on downregulation of Dot1a and Af9 and upregulation of ET-1 in NRK-52E cells. (A–C ) NRK-52E cells were treated with ethanol as vehicle control (0) or 10–100 nM aldosterone (Aldo) and analyzed by MTT assay for cell viability at 24 h (**A**), 48 h (**B**), and 72 h (**C**). n = 3. In all cases, *P<0.05 vs. CT. **(D–F)** NRK-52E cells were treated as in **A** for 24 h, and analyzed by RT-PCR for mRNA expression of the genes indicated. n = 3–6. (**G–H**) Representative IF images showing Af9 and ET-1 expression. (**I**) Representative IBs showing expression of Af9 and ET-1. Additional CT and aldosterone-treated samples were analyzed and shown in Fig. 8. Note, all samples received the same amount of ethanol as vehicle control. (**J–K**) The intensity of each band in **I** was analyzed as in Fig. 5E–F. n = 3–6. In all cases, *P<0.05 vs. CT. #P<0.05 as shown. For simplicity, comparison between aldosterone-treated cells was limited to the adjacent two doses.

To verify our results, we chose rat kidney epithelial NRK-52E cells, which have been shown to respond to aldosterone [48], [49], [50]. Cell viability assays revealed that all concentrations of aldosterone ranging from 0.01 to 100 nM tested had little cytotoxic or proliferative effects on NRK-52E cells at the 24, 48, and 72 h time points examined (Fig. 7A–C).

To determine if aldosterone regulates Dot1a, Af9, and ET-1 in a dose-dependent manner, we cultured NRK-52E cells in normal medium (CT) or treated with 10, 25, 50, or 100 nM aldosterone for 24 h. The volumes of the vehicle (ethanol) added to the medium were equal among the treatments. While 10 and 25 nM aldosterone were almost equally effective and sufficient in inhibiting Dot1a, aldosterone at 25 nM was more effective in downregulating Af9 and in stimulating ET-1 mRNA expression than at 10 nM. Further increases in aldosterone dose to 50 and 100 nM had little additional effects on Dot1a mRNA expression. However, both Af9 and ET-1 mRNA levels were even lower in the cells treated with aldosterone at 50 nM than at 25 nM (Fig. 7, D–F). Further increasing aldosterone dose to 100 nM had little effect on the mRNA expression of these two genes. As shown in Fig. 7, G–K, IF and IB revealed a progressive reduction in Af9 protein levels with increasing amounts of aldosterone. ET-1 protein expression showed a complex response to aldosterone concentrations, with progressive increase from 0 to 25 nM, little effect from 25 nM to 50 nM, and a significant reduction from 50 nM to 100 nM. Taken together, these data suggest that aldosterone regulates these genes in a dose-dependent manner, although the dosage effect was much less pronounced at the mRNA level for Dot1a and ET-1, and more complex for ET-1 protein under the conditions tested.

**Figure 8:**
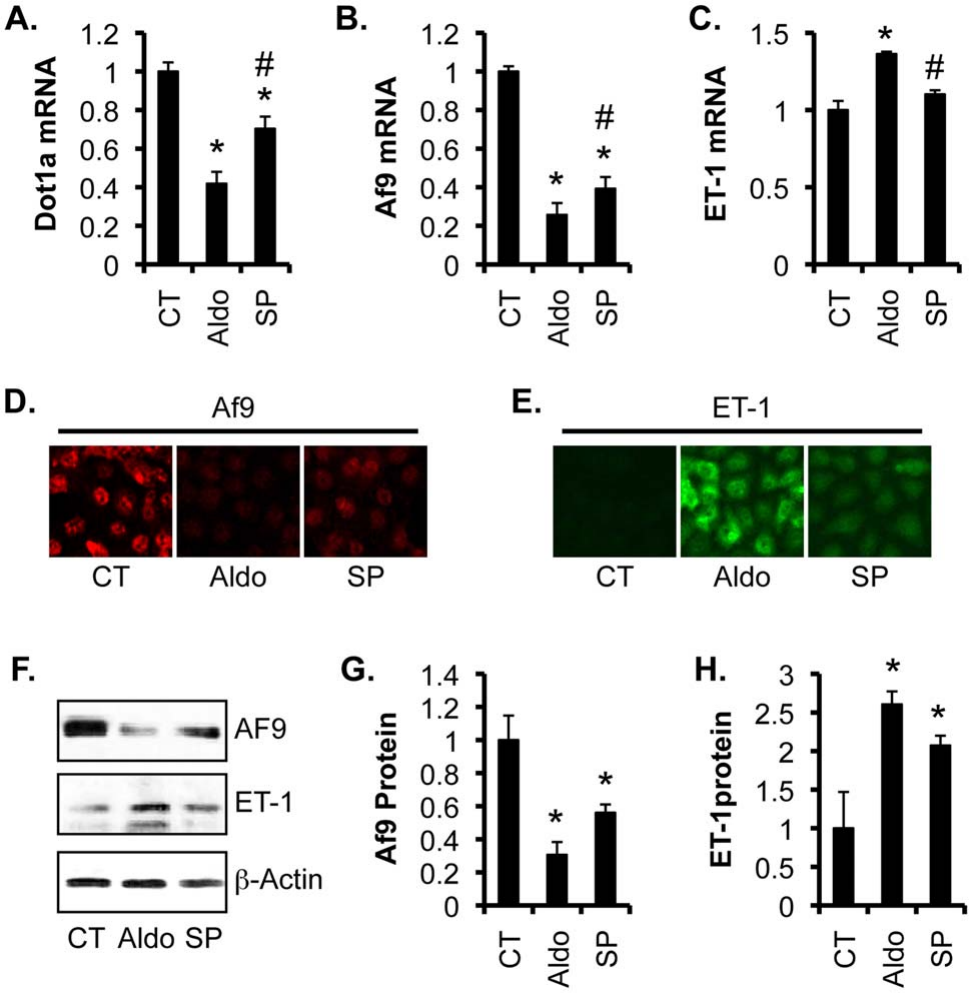
Spironolactone partially reversed the effects of aldosterone on expression of Dot1a, Af9, and ET-1 in NRK-52E cells. (**A–C**) NRK-52E cells were treated with vehicle as control (CT), 50 nM aldosterone (Aldo), or 50 nM aldosterone plus 1-h pretreatment with 100 nM spironolactone (SP) for 24 hours, and analyzed as in Fig. 7**D–F**. n = 3. (**D–E**) Representative IF images showing Af9 and ET-1 expression. (**F**) Representative IBs showing expression of Af9 and ET-1. (**G–H**) The intensity of each band in **F** was analyzed as in Fig. 5E–F. n = 3. In all cases, *P<0.05 vs. CT. #P<0.05 vs. Aldo.

To evaluate the effect of spironolactone on Dot1a, Af9, and ET-1 expression, NRK-52E cells were treated with vehicle as control, 50 nM aldosterone, or 50 nM aldosterone +100 nM spironolactone for 24 h. Compared to vehicle control, aldosterone significantly decreased Dot1a and Af9 and increased ET-1 mRNA levels to 42%, 26%, and 137% of control, respectively (Fig. 8, A–C). More pronounced effects were observed at the protein level. IF and IB revealed robust Af9 expression and minimal ET-1 expression in control cells. Aldosterone reduced Af9 and increased ET-1 protein synthesis (Fig. 8, D–F). Quantification of the IBs revealed that aldosterone treatment markedly decreased Af9 and increased ET-1 protein abundance to 30% and 260% of control, respectively. In all cases, the aldosterone effect was attenuated by prior treatment of the cells with spironolactone (Fig. 8, G–H). As stated above, the unavailability of an antibody recognizing rat Dot1a made it impractical to determine Dot1a protein expression in these experiments.

### Overexpression of Dot1a and Af9 Reverses Aldosterone-induced ET-1 Expression in NRK-52E Cells

**Figure 9:**
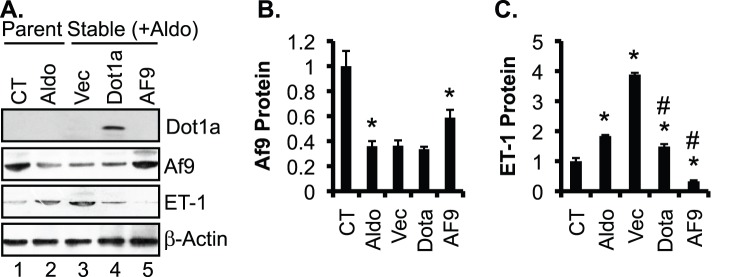
Overexpression of Dot1a and Af9 reverses aldosterone-induced ET-1 expression in NRK-52E cells. NRK-52E cells were treated with vehicle as control (CT), or treated with 50 nM aldosterone (Aldo) for 24 h, and were analyzed in parallel with stably transfected NRK-52E cell lines. These cell lines had integrated pcDNA3.1 as vector control (Vec), or its derivatives expressing untagged mouse Dot1a (Dot1a) or Af9 (Af9) and were treated with 50 nM aldosterone (Aldo) for 24 h. (**A**) Representative IBs showing expression of Dot1a, Af9, and ET-1, with β-actin as internal control. Note, the anti-Dot1a antibody detected the overexpressed mouse Dot1a, but apparently not the endogenous rat Dot1a, possibly due to its poor reactivity with rat Dot1a, low level of rat Dot1a, or both. (**B–C**) The intensity of each band was analyzed as in Fig. 5E–F. n = 3–6. In all cases, *P<0.05 vs. CT. #P<0.05 vs. Vec.

Whether overexpression of Dot1a and Af9 reverses aldosterone effects on ET-1 induction remain unknown. Accordingly, we generated three stable NRK-52E cell lines harboring an empty vector as control, or a construct encoding untagged Af9 or Dot1a. These were cultured in the presence of G418 (800 µg/ml, to maintain the selection for the integrated plasmids) and 50 nM aldosterone for 24 h. Parent NRK-52E cells with or without 50 nM aldosterone treatment for 24 h were also included as controls. IB was performed to evaluate protein expression of Dot1a, Af9, and ET-1. The anti-Dot1l antibody, which has been used by others [51], detected the overexpressed mouse Dot1a in the Dot1a-transfectants, but not the endogenous rat Dot1a in the parental or transfected cells (Fig. 9A). Failure to detect the endogenous Dot1a might result from the poor reactivity of the antibody towards the rat Dot1a, low level of the endogenous Dot1a, or both. Compared to parent cells grown in normal medium, parent cells treated with aldosterone had significantly decreased Af9 and increased ET-1 expression. Transfection of the empty vector or Dot1a had little impact on Af9 expression, relative to aldosterone treated parent cells (compare lanes 3 and 4 with lane 2, Fig. 9A). Transfection of the Af9 construct partially reversed aldosterone effect on Af9 expression. The induction of ET-1 by aldosterone was more pronounced in the cells stably transfected with the empty vector, which was presumably due to the addition of G418 to the media for the stable cell lines. However, the aldosterone-mediated induction of ET-1 was markedly blocked by overexpression of either Dot1a or Af9 (compare lanes 4 and 5 with lane 2, Fig. 9A). ET-1 protein levels were significantly decreased from 388% in vector-transfected cells to 148% in Dot1a-transfected cells and to 33% in Af9-transfected cells, suggesting that overexpression of Dot1a and Af9 can effectively reverse aldosterone-mediated induction of ET-1 protein expression (Fig. 9, A–C).

### Aldosterone Reduces Binding of Dot1a and H3 K79 Methylation at the *Edn-1* 5′ Regulatory Region and Upregulates ET-1 mRNA Expression in IMCD3 Cells

**Figure 10:**
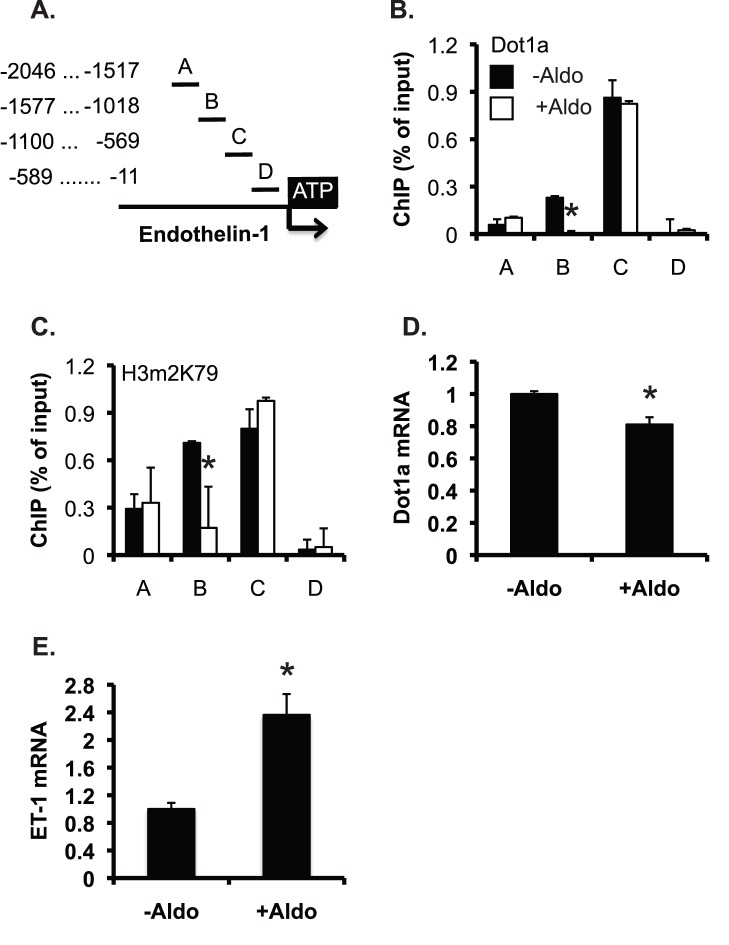
Aldosterone reduces binding of Dot1a and H3 K79 methylation at the *Edn-1* 5′ regulatory region and upregulates ET-1 mRNA expression in IMCD3 cells. (**A**) Diagram of the 5′ regulatory region of *Edn-1*. (**B–C**) Chromatin immunoprecipitation showing Dot1a and H3m2K79 binding in the 5′ regulatory region of *Edn-1.* Chromatin from IMCD3 cells treated with ethanol as vehicle control (-Aldo) or 1 µM aldosterone (+Aldo) for 24 h was immunoprecipitated by the rabbit antibodies specific for Dot1a (**B**) and H3m2K79 (**C**), followed by real-time qPCR with primers amplifying subregions A-D shown in **A**. Relative ChIP efficiency was defined as the immunoprecipitated amount of materials present as compared to that of the initial input sample. *: P<0.05 vs. –Aldo within the same subregion. (**D–E**) IMCD3 cells were treated as in **B–C** and analyzed by real-time RT-qPCR for mRNA expression of Dot1a (**D**) and ET-1 (**E**). n = 2. *: P<0.05 vs. –Aldo.

To investigate if aldosterone modulates H3 K79 methylation at *Edn-1* 5′ flanking region, IMCD3 cells were treated with ethanol as vehicle control or 1 µM aldosterone for 24 h, and analyzed by chromatin immunoprecipitation (ChIP) assay coupled with real-time qPCR, as we previously reported [31], [32], [33], [36]. The 2.0-kb upstream region of *Edn-1* was divided into 4 subregions named as A-D, respectively. Each of them was amplified with a pair of primers (Fig. 10A). Various levels of Dot1a binding with the strongest binding in C and little binding in D were observed in control cells. Aldosterone significantly impaired Dot1a association with B, but had little impact in all other subregions (Fig. 10B). A very similar binding profile for H3m2K79 was also observed (Fig. 10C). Real-time RT-qPCR revealed that aldosterone significantly decreased Dot1a mRNA to 80% of control and increased ET-1 mRNA to 236% of control (Fig. 10, D-E). Collectively, aldosterone stimulates ET-1 mRNA expression, possibly by inhibiting Dot1a mRNA and thus H3m2K79 binding at *Edn-1* 5′ regulatory region in IMCD3 cells.

### *Dot1l* Deficiency Leads to Upregulation of ET-1 in Mouse Kidney

**Figure 11:**
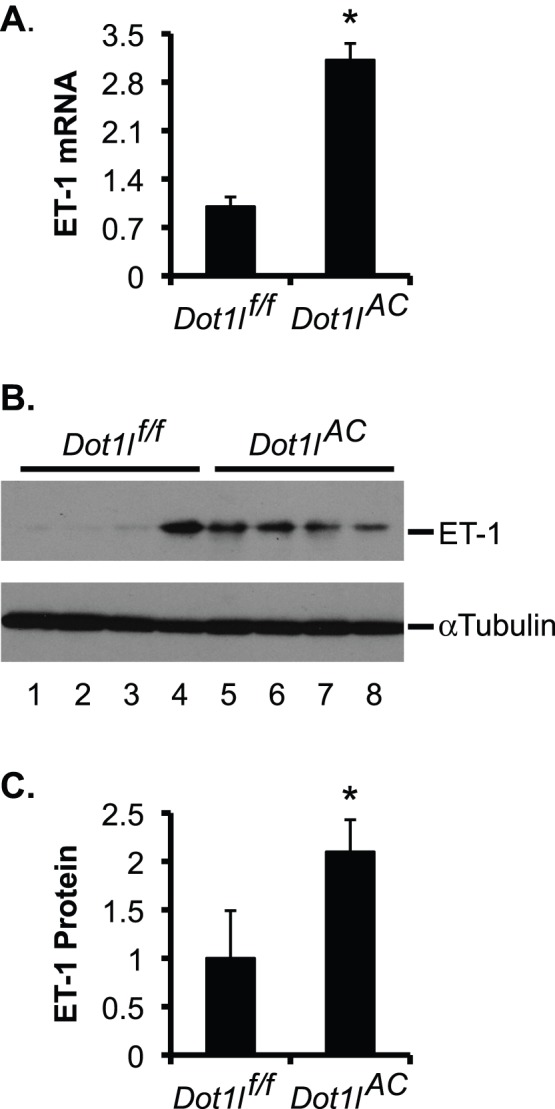
Inactivation of *Dot1l* increases ET-1 expression at mRNA and protein levels in mouse kidney. (**A**) Real-time RT-qPCR for expression of ET-1 in kidneys of *Dot1l^f/f^* and *Dot1l^AC^* mice, with β-actin as internal control. n = 4 mice/group. (**B**) As in **A**, IB for ET-1 expression, with β-actin as internal control. (**C**) The intensity of each band in **B** was analyzed as in Fig. 5E–F. In all cases, *P<0.05 vs. *Dot1l^f/f^* mice.

We have developed a new *Dot1l* conditional knockout mouse model (*Dot1l^AC^*), which lacks the vast majority, if not all, of Dot1a function including the methyltransferase activity and the ability to interact with Af9 in Aqp2-expressing renal principal cells (Wu et al, 2011 ASN abstract 21278, and Wu et al, 2011 ASN abstract 20636). If Dot1a-mediated repression of *Edn-1* occurs in the principal cells, deletion of *Dot1l* would lead to upregulation of ET-1 expression. Consistently, real-time RT-qPCR (n = 6 mice/group) and IB (n = 4 mice/group) uncovered a >3-fold higher ET-1 mRNA and doubled ET-1 protein expression in *Dot1l^AC^* kidney than in control (*Dot1l^f/f^*) (Fig. 11A–C). These data indicate that loss of *Dot1l* function enhances ET-1 expression at both mRNA and protein levels in mouse kidney, and suggest that Dot1a represses ET-1 transcription in vitro and in vivo.

### *Dot1l^AC^* vs. *Dot1l^f/f^* Mice had Reduced Binding of Dot1a and H3 K79 Dimethylation at the *Edn-1* 5′ Regulatory Region

**Figure 12:**
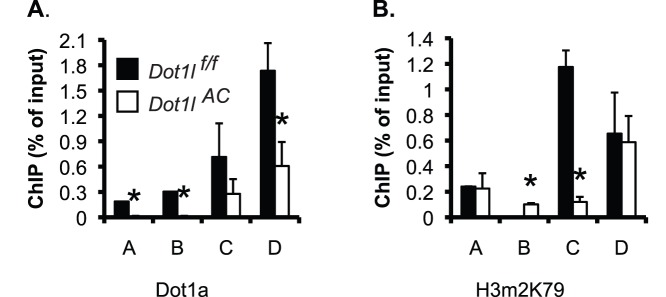
Dot1a and H3m2K79 bind at specific subregions of *Edn-1* 5′ regulatory region in mouse kidneys. (**A–B**) As in Fig. 10B-C except that chromatin was prepared from *Dot1l^f/f^* and *Dot1l^AC^* mice on the normal Na^+^ pellet diet (n = 6 mice/group) *: P<0.05 vs. *Dot1l^f/f^* within the same subregion.

To investigate if Dot1a represses *Edn-1* transcription by promoting H3 K79 methylation at *Edn-1* 5′ flanking region in vivo, we performed ChIP assay coupled with real-time qPCR and detected Dot1a binding in all of the 4 subregions of the *Edn-1* 5′ flanking in *Dot1l^f/f^* mice. The occupancy of Dot1a in these subregions was significantly impaired in *Dot1l^AC^* mice. In particular, the ChIP signal with the anti-Dot1a became undetectable in subregions A and B in *Dot1l^AC^* mice (Fig. 12A). The impairment of Dot1a binding is consistent with the fact that disruption of *Dot1l* upon Cre recombinase expression driven by the Aqp2 promoter is restricted to Aqp2^+^ cells, rather than taking place in the whole kidney cell population. A similar binding profile for H3m2K79 was observed, with exception of subregion B, where the ChIP with the anti-H3m2K79 yielded little or very weak signals in *Dot1l^f/f^* and *Dot1l^AC^* mice (Fig. 12B). These observations further demonstrate the specificity of Dot1a and H3m2K79 antibodies that have been used by others and us [31], [33], [36], [51]. Furthermore, ChIP with normal rabbit IgG yielded barely detectable background binding (data not shown). It should be noted that although Dot1a and H3m2K79 exhibited little binding in D in control IMCD3 cells (Fig. 10, B–C), they bound D strongly in *Dot1l^f/f^* mice (Fig. 12). The difference in the binding of Dot1a and H3m2K79 with D between these two experiments is probably attributable to the purity in cell population, which is expected to be much higher in the homogenous IMCD3 cells than in the heterogeneous mouse kidney. In brief, Dot1l and H3m2K79 reside at particular subregions of *Edn-1* promoter. Deletion of *Dot1l* presumably eliminates the occupancy of Dot1a and H3m2K79 in these subregions in the Aqp2^+^ cells in the mutant kidney. Such elimination can be inferred from the observed overall reduction of the corresponding ChIP signals using the whole mutant kidney. Therefore, Dot1la downregulates *Edn-1* mRNA expression, at least partially by modulating H3 m2K79 at the *Edn-1* promoter.

## Discussion

Despite much interest in the role of aldosterone and ET-1 in the pathogenesis of DN, the underlying molecular players connecting the actions of these two hormones are not fully elucidated. In the current study, we describe the following new findings: 1) The increased aldosterone and ET-1 synthesis in the diabetic kidney tissue are associated with decreased expression of Dot1a and Af9; 2) Spironolactone decreased ET-1 and increased Dot1a and Af9 expression, which may be contributory to the amelioration of the diabetic phenotype; 3) Spironolactone induces a similar effect on expression of ET-1, Dot1a, and Af9 in NRK-52E cells; 4) Dot1a and H3m2K79 are specifically associated with the 5′ regulatory region of *Edn-1* in IMCD3 cells and in mouse kidney; and 5) Genetic inactivation of *Dot1l* in the Aqp2-expressing principal cells caused a general reduction of Dot1a and H3m2K79 binding at the 5′ regulatory region of *Edn-1* and a significant increase in ET-1 mRNA and protein expression. Taken together, our data strongly support the hypothesis that Dot1a and Af9 repress *Edn-1* expression at both mRNA and protein levels in vitro and in vivo; elevated aldosterone contributes to kidney injury, in part by downregulating Dot1a and Af9 to enhance *Edn-1* transcription. Spironolactone executes its reno-protective effect, possibly by restoring Dot1a-Af9-mediated repression to lower *Edn-1* transcription.

Fujisawa et al reported that STZ (65 mg/kg, single intraperitoneal injection) induced collagen deposition in glomerular, tubulointerstitial, and perivascular areas in rat kidney three weeks after the STZ treatment. Administration of spironolactone (50 mg/kg/day sc) after STZ injection for three weeks completely ameliorated the collagen deposition [6]. Using a very similar protocol with STZ administered by intravenous injection (55 mg/kg) and spironolactone by gavage (50 mg/kg per day) for three weeks, Taira et al also reported that STZ significantly increased urinary protein excretion and collagen deposition in glomerular and tubulointerstitial areas in the kidney, and SP attenuated these pathological changes [42] In this study, we also applied a single, intraperitoneal dose of STZ (60 mg/kg body weight). In the SP rats, spironolactone treatment was not immediately started following STZ administration. Instead, the rats were allowed to develop kidney damage for 4 weeks and then left untreated (STZ group) or treated with spironolactone (40 mg/kg/day) for 4 more weeks. In this way, the induction of kidney injury was maintained for 8 weeks in STZ groups and interfered with by spironolactone for the last 4 weeks in the SP group. The ameliorated phenotype in the SP vs. STZ rats suggests that spironolactone may be able to reverse the pathological changes even after it has been initiated.

In our study, spironolactone increased [aldosterone] in the kidney tissue, but not in the plasma (Fig. 1, H & I). Inhibition of mineralocorticoid receptor may thus induce a compensatory mechanism that stimulates aldosterone production and/or inhibits its metabolism within the kidney. The elevated [aldosterone] in turn presumably promotes ET-1 production in aldosterone-responsive cells, which include the Aqp2-expressing principal cells (PCs) in the collecting duct [52], [53]. Similarly, deletion of *Dot1l* in these PCs using an Aqp2:Cre line [39] not only abolished Dot1a-mediated H3m2K79 in these cells (Wu et al, 2011 ASN abstract 21278, and Wu et al, 2011 ASN abstract 20636), but also significantly elevated ET-1 mRNA and protein expression in whole kidney. We used the same anti-ET-1 antibody in Fig. 5 to perform IB, IF and IHC of *Dot1l^f/f^* and *Dot1l^AC^* kidneys. Although the antibody recognized mouse ET-1 in IB (Fig. 11B), it failed to specifically detect mouse ET-1 in IF and IHC (data not shown). Similar technical challenges may also have hindered others from demonstrating the disruption of ET-1 synthesis in PCs in CD ET-1 KO mice, which were generated with a different *Aqp2Cre* line [52].

Aldosterone has been shown to induce ET-1 expression in vascular smooth muscle cells [54], cardiomyocytes [55], mouse inner medullary collecting duct IMCD3 cells, acutely isolated rat inner medullary collecting duct cells ex vivo, and rat inner medulla in vivo [47], [56]. In IMCD3 cells, aldosterone promotes nuclear translocation and binding of mineralocorticoid receptor and glucocorticoid receptor to the *Edn-1* hormone response elements in a dose-dependent manner. Administration of spironolactone or RU486 completely blocks aldosterone induction of ET-1 mRNA, suggesting that both mineralo- and glucocorticoid receptors are functionally required for the aldosterone-mediated induction of *Edn-1*. In addition, DNA methylation and histone H3 K4 methylation have been shown to regulate the tissue-specific expression [57] and aldosterone-mediated induction of *Edn-1* [56]. It should be stressed that increased ET-1 in the diabetic kidney may also be aggravated by the activation of multiple cytokines and growth factors such as TGF-β [58].

Upregulation of ET-1 by elevated aldosterone or by *Dot1l* deletion not only reinforces the notion that *Edn-1* is transcriptionally regulated by the aldosterone signaling network that includes Dot1a and Af9, but also suggests the underlying physiological significance. The increased ET-1 presumably functions as an autocrine to induce local injury. ET-1 may also play its detrimental role in a paracrine manner, as described by others [8]. The histological changes in glomeruli, in which expression of Af9 and ET-1 was significantly lower than in tubular epithelia (Fig. 5A), might partially result from the elevated ET-1 functioning in a paracrine manner.

Nevertheless, further studies using animal models deficient in *Dot1l*, *Af9*, and *Edn-1* are required to conclusively demonstrate the role of these players in the spironolactone-mediated improvement of kidney damage in STZ-induced diabetic animals. It would also be interesting to know if similar mechanisms are applicable to other hormones, stimuli, and pharmacological agents that are known to directly interfere with the spatial, temporal, and quantitative expression of *Edn-1*. In addition, Dot1a and Af9 inhibitors, which currently are not available, would also need to be tested for reno-protective effects.

In conclusion, spironolactone treatment decreased urinary albumin excretion and lessened glomerulosclerosis and tubulointerstitial injury. The underlying molecular mechanism involves restoration of Dot1a-Af9 repression, leading to a reduction of ET-1 synthesis. In this regard, Dot1a and Af9 can be considered as potential therapeutic targets for the management of DN.
